# Physiology as a Lingua Franca for Clinical Machine Learning

**DOI:** 10.1016/j.patter.2020.100017

**Published:** 2020-05-08

**Authors:** Gopal P. Sarma, Erik Reinertsen, the ML4CVD Group, Aaron Aguirre, Chris Anderson, Puneet Batra, Seung-Hoan Choi, Paolo Di Achille, Nathaniel Diamant, Patrick Ellinor, Connor Emdin, Akl C. Fahed, Samuel Friedman, Lia Harrington, Jennifer E. Ho, Amit V. Khera, Shaan Khurshid, Marcus Klarqvist, Steve Lubitz, Anthony Philippakis, James Pirruccello, Christopher Reeder, Collin Stultz, Brandon Westover

**Affiliations:** 1 Broad Institute of MIT and Harvard, Cambridge, MA, USA; 2 Massachusetts General Hospital, Boston, MA, USA; 3 Massachusetts Institute of Technology, Cambridge, MA, USA

## Abstract

The intersection of medicine and machine learning (ML) has the potential to transform healthcare. We describe how physiology, a foundational discipline of medical training and practice with a rich quantitative history, could serve as a starting point for the development of a common language between clinicians and ML experts, thereby accelerating real-world impact.

## Main Text

Many editorials and press releases herald the coming transformation of medicine via machine learning (ML). Papers report assessing diabetic retinopathy from retinal scans,^1^ identifying arrhythmias from electrocardiograms (ECGs),^2^ and predicting outcomes from electronic medical records (EMRs).^3^ Nonetheless, obstacles ranging from poorly integrated EMRs to the limited availability and biased nature of clinical datasets for ML research have prevented ML from having a widespread impact on medicine.^4^ Moreover, underpinning these technical and infrastructural obstacles lie a more nuanced set of issues related to the deep cultural differences between the ML and clinical communities.

One oft-cited cultural and technical concern from clinicians is the “black box” nature of ML—the disconnect between medical domain knowledge and an opaque algorithm.^5^ While medicine has made use of algorithms and statistics for decades, the cases and input variables used have been carefully vetted and universally acknowledged as important. In contrast, modern ML involves building models with vast and noisy training examples, input variables, and parameters—often larger than those used in the past by several orders of magnitude. Consequently, the medical establishment has reacted to the complexity of modern ML with understandable hesitation. In particular, the interpretability of ML models in medicine is critical, given that the model outputs will shape decisions related to people’s health and wellness.

Conversely, the complexities of biology, medicine, and healthcare are themselves a black box to ML practitioners. In contrast to carefully curated ML benchmarks such as ImageNet, clinical datasets are extremely noisy, biased, and generated by a complex set of interactions among patients, providers, and healthcare systems, all of which can make medicine as a whole seem unapproachable. This prevents ML scientists from readily integrating the considerable value of medical domain expertise.

How can we bridge this gap and find common ground? We believe that the *physiology of organ systems* provides a starting point for the emergence of a common language that leverages both sets of expertise, particularly in the context of the subfield of representation learning, which we discuss below. We use a broad interpretation of physiology to denote existing bodies of knowledge pertaining to biological tissues, anatomy, and disease processes around which ML models could be oriented. Many advances in physiology have come from quantitative disciplines such as mathematics, statistics, physics, computer science, and engineering, fields that today are core drivers of ML research. Furthermore, physiology is the conceptual basis for medical education and practice. Developing this lingua franca between ML and medicine will reduce collaborative friction, align novel methods with pragmatic needs, and maximize contributions from both communities to the research, development, and implementation of clinical ML.

A subdiscipline of ML well positioned to bridge the gap with medicine is known as “representation learning.” Representation learning is concerned with techniques to learn useful features of data and disentangle sources of variation, while retaining the essential elements of the underlying generative processes.^6^ What constitutes a “meaningful representation” varies by domain and is often difficult to precisely define. Indeed, entire workshops and conferences have been devoted to exploring this question in both technical and philosophical terms (see, for example, the International Conference on Learning Representations or the NeurIPS workshop “Learning Meaningful Representations of Life”). For example, imagine an ML system trained to predict the trajectory of a projectile in flight, such as an apple falling from a tree. Given enough training data such as high-resolution video of apples falling, we expect modern neural networks to perform quite well on this task. However, what if a learning system was able to recover basic concepts from Newtonian physics? Learning the dynamics of projectile motion, with measures of velocity, angle of launch, gravity, etc., would elevate an accurate prediction to a meaningful representation. Or more modestly, what if our ML system was built using prior knowledge of the world rather than being a tabula rasa?

In this spirit, we propose that physiological models of organ systems—constructed from high-dimensional clinical data such as laboratory tests, vital signs, imaging, etc.—could serve as meaningful representations of clinical data. Concepts and intuition from physiology provide the basis for a common language that bridges the knowledge, technical expertise, and intellectual culture of both the ML and clinical communities. To illustrate what representation learning of physiology might look like in a clinical application, consider the opposite extreme of building systems to predict outcomes *without* modeling the underlying physiological processes that give rise to the data. Such models can be quite impressive, both in their technical infrastructure as well as objective measures of performance. Recent work with EMR data, for example, has shown that massive neural networks can ingest entire time series of inpatient hospital stays and output the exact set of ICD-10 diagnostic codes with near-perfect accuracy.^3^ These systems may play a role in the future of healthcare by streamlining operations and billing, thus reducing administrative overhead. However, such models do not require, nor do they leverage, knowledge of biology, medicine, or healthcare. Medical professionals might be reasonably apprehensive, therefore, about deploying such models for clinical, rather than purely logistical, purposes.

On the other end of the spectrum, imagine systems designed with two complementary goals: to make clinically useful predictions and, in addition, to produce physiologically interpretable output, possibly as a secondary task. For example, researchers recently trained a recurrent neural network to predict whether or not patients would develop acute kidney injury (AKI) during a hospital stay.^7^ Crucially, the system also performed an auxiliary task—to predict the final lab values of parameters relevant to renal physiology (specifically, the serum creatinine, urea nitrogen, sodium, potassium, chloride, calcium, and phosphate). Predicting both an outcome of interest (similar to the EHR results discussed above) and physiologically relevant variables is a first step toward model output that is both actionable and clinically interpretable. Although efforts to develop and implement ML systems at the point of care are still nascent, one can imagine advantages to systems that operate in a manner more readily understandable by humans. Such an attribute could enable identification of failure modes, i.e., patients or situations in which the model performs poorly. For example, if the AKI prediction was less accurate when the simultaneously predicted lab values were inconsistent with the primary outcome (e.g., when the system predicts AKI and normal lab values or predicts no AKI and abnormal lab values), the ML team could revise the model to address these edge cases, and the clinical team could give the model prediction less weight relative to the other information they use to make decisions.

Physiologically interpretable models, particularly those based on organ systems, are an ideal abstraction to integrate clinical expertise and ML because they closely parallel the mental models of how physicians learn and practice medicine. First, physicians gather and organize information about patients in organ-system-based schemas. Second, as a result, most medical schools around the world have shifted the preclinical curriculum away from traditional academic disciplines such as biochemistry, cell biology, or immunology toward organ-system-based blocks, such as the cardiovascular system, pulmonary system, nervous system, etc. Third, as a result of these two factors, models that are broadly organized around physiology and organ systems may play a role in increasing the transparency and interpretability of ML systems integrated into a healthcare setting.

How can we operationalize building physiological models that are geared toward clinical impact? As we discussed above, the notion of a meaningful representation rarely has a precise definition and often requires considerable expertise from multiple domains to realize in practice. A critical first step is to create interdisciplinary teams that integrate ML and clinical expertise in an environment that encourages the development of shared concepts. We are experimenting with such an approach through the ML for Cardiovascular Disease initiative (ML4CVD), a collaboration between the Broad Institute, Massachusetts General Hospital, and Massachusetts Institute of Technology. ML4CVD builds physiological models of the cardiovascular system by harnessing multi-modal data, such as the ECG, echocardiogram, and cardiac magnetic resonance imaging. We develop configurable software tooling to enable researchers to iterate on training families of ML models that are optimized for learning representations (specifically, autoencoders) in order to make clinically meaningful classifications and predictions.

In the research setting, this approach has provided us with rich representations of cardiovascular phenotypes that enable discovery of new genetic risk factors (Friedman et al., 2019, NeurIPS, conference). In a clinical setting, such models could ultimately augment, rather than replace, clinical reasoning in the context of missing or pending lab tests or imaging, in expensive and sophisticated tests that are not universally available, or in resource-poor settings lacking specialist expertise. To encourage cross-disciplinary dialogue and collaboration, we utilize several organizational strategies: regular team meetings to discuss operational details, seminars that blend medical and technical content, and team-building challenges such as requiring clinicians to periodically present ML results and engineers to present clinical results. Our belief is that through trial and error and iterative discussion, such teams will ultimately converge on the “right” set of representations to advance machine learning applications in clinical settings.

Despite considerable excitement and a steady stream of impressive research results, the impact of ML on healthcare is in its early stages. Unlike current algorithms that have been productively deployed in clinical settings, such as rule-based ECG classification or clinical risk scores, modern ML distinguishes itself with the size of the datasets typically used to train models, the number of variables involved, and the complexity of the interactions between those variables. Physiology, a discipline that is the basis of medical education and decision making, provides a rich set of concepts to inform the development of ML models that can be vetted and transparently understood by clinicians. To facilitate this integration, we propose that a *default expectation* should be for clinically relevant ML models to predict physiologically relevant variables, even at the expense of prediction accuracy. Identifying physiological considerations and understanding the trade-offs between performance and physiological interpretability is a complex and iterative process that requires sustained engagement between the ML and clinical communities. The development of a shared vocabulary will be a critical enabler of this growth process, which will ultimately have a transformative impact on the diagnosis and treatment of disease.
